# Balancing uncertainty and proactivity in care seeking for hepatitis C: qualitative research with participants enrolled in a treatment trial in Ho Chi Minh City, Vietnam

**DOI:** 10.1080/17482631.2022.2126602

**Published:** 2022-09-26

**Authors:** My Nguyen Le Thao, Yen Nguyen Thi Hong, Thuan Dang Trong, Nguyen Thanh Dung, Jeremy Day, Le Thanh Phuong, Evelyne Kestelyn, Nguyen Van Vinh Chau, Hung Le Manh, Jennifer Ilo Van Nuil

**Affiliations:** a Oxford University Clinical Research Unit, Ho Chi Minh City, Vietnam; b Hospital for Tropical Diseases, Ho Chi Minh City, Vietnam; c Nuffield Department of Medicine, University of Oxford, Oxford, UK

**Keywords:** DAA treatment, viral hepatitis, clinical trial, care seeking, qualitative, Vietnam

## Abstract

**Purpose:**

Direct acting antiviral treatment to cure hepatitis C virus (HCV) is becoming more accessible yet the experiences of those accessing care and treatment and the contexts under which care seeking takes place are largely unknown in low- and middle-income countries. These experiences are important for insight into the challenges people encounter and the support/structures they utilize. The study objective was to explore the experiences of care seeking and treatment for participants enrolled in a clinical trial in Ho Chi Minh City, Vietnam.

**Methods:**

We used in-depth interviews, home visits, mobile interviews, at both the clinic and in the home as we explored how participants experienced health and illness within their social worlds over time.

**Results:**

We enrolled 20 participants, of whom 20 completed the first interview, 16 the second, and 18 completed the last interview. Findings explore four themes: (1) navigating uncertainty, (2) proactivity in the face of challenges, (3) living in fear with faith, and (4) dynamic support systems.

**Conclusions:**

Understanding how participants envision and act upon their lived experiences can help to develop public health programmes that effectively address barriers and promote access to care and treatment for people with HCV in Vietnam.

## 1. Main text introduction

Viral hepatitis, including hepatitis B and C, is a major public health problem requiring an urgent global response. Worldwide, approximately 71 million people are living with chronic hepatitis C virus (HCV) although this number is likely increasing despite the existence of curative treatments including direct acting antiviral drugs (DAAs; Cheemerla & Balakrishnan, 2021). Since the introduction of DAAs in 2014, less than 10% of people with HCV accessed treatment and were successfully cured (Cheemerla & Balakrishnan, 2021; World Health Organization). 2020 Around 80% of the global HCV burden is from low and middle-income countries (LMICs), with the majority of cases recorded in South Asia, East Asia, Southeast Asia, the Middle East, and North Africa (Dhiman, 2014; Hanafiah et al., 2013; Jayasekera et al., 2014). In 2016, LMICs accounted for 75% of HCV cases worldwide, even though only 8% of people living with HCV in low income countries are thought to have been diagnosed (WHO, 2018). Access to DAAs in LMICs, at the time of the study, was still limited despite it being an effective treatment of chronic HCV therefore, it is crucial to understand the make-up of local epidemics, especially in light of the 2030 World Health Organization’s (WHO) hepatitis elimination goals (World Health Organization, 2016a). The ways in which people experience viral hepatitis and ignite their care-seeking process are highly varied across different groups, and tied deeply to the context where social, cultural and economical factors simultaneously influence their decisions (Mattingly et al., 2019; Wait et al., 2016). The scale up of DAA treatments will be determined by both patients’ efforts to manouver along their “situated” pathway to cure and the responsibility from the government and wider public to understand and address care seeking challenges.

Several qualitative studies have been conducted to obtain forms of knowledge about the care seeking experiences of patients with viral hepatitis and/or liver diseases, both before and after the introduction of DAAs. In high income countries before DAAs access, significant levels of stigma and lack of support in care-seeking, and an overall reduction in quality of life upon diagnosis and beyond were reported (Dowsett et al., 2017). Living with chronic HCV infection has also been documented to have periods of high stress and loss of social support after disclosure to friends and family (Miller et al., 2012). With the introduction of DAAs, participants reported experiencing decreased perceptions of stigma often resulting in increased disclosure (Madden et al., 2018; Richmond et al., 2018; Wright et al., 2019). In Australia, being cured brought people with an injecting history a sense of freedom as they could detach from the past chapters of their life; others reflected that being cured meant they could consider and plan for their future in a promising way (Richmond et al., 2018).

In LMICs, the experiences reported revolved around care and treatment access, uncertainty, and stigma. In Cameroon, for example, the experiences of viral hepatitis diagnosis resulted in deep financial distress for those trying to access care, as well as uncertainty (Chabrol et al., 2019). Similarly, in Burkina Faso, people living with hepatitis B reported a state of uncertainty, while also noting that the feeling of “not knowing” was in fact a stimulus for them to confront and reimagine both their past and future (Giles-Vernick & Hejoaka, 2020). Even if patients could afford care and treatment, being stigmatized or self-stigmatized often still remains a barrier for people with hepatitis to seek care and treatment (World Health Organization, 2016b). For example, in studies from Egypt, people living with HCV, like other blood borne diseases, experienced stigma causing them to “feel dirty” about themselves and ultimately break social ties as they often stopped meeting friends and family to avoid potential stigma, or because they thought that other people simply did not enjoy being close to those who were sick (Soltan et al., 2018). However, the situation in Rwanda was reported to be the opposite where researchers found lack of stigma among people with HCV and their family members. Alternatively, people proactively formed support groups, including people who were taking treatment at the same facility, to encourage each other to adhere to treatment (Serumondo et al., 2020). In Vietnam, there have been a few qualitative studies on the care seeking experiences after DAAs became available in 2016. However, the availability of DAAs to the general population was limited due to its high cost that were calculated to reach 200 million VND for treatment for one person (approximately 8500 USD; Vietnam Assocation of Preventive Medicine, 2019). In 2018, the government of Vietnam approved that DAAs would be 50% covered by health insurance. Unfortunately, it was only applicable for people with health insurance registered at the national and provincial level hospitals, which meant that people care seeking at lower level hospitals, such as district hospitals or healthcare centres, were excluded (World Health Organization, 2018). Further in a recent study by Rapoud et al. (2020) in Hai Phong, the authors scrutinized the effectiveness of the care strategy with DAAs. They suggest that for the underserved and higher-risk populations (e.g., PWID), a simplified integrated care model would result in a higher cure rate of HCV. Additionally, treatment adherence and prevention of reinfection were noted as decisive factors to eliminate HCV (Rapoud et al., 2020).

In this study, we conducted qualitative research with a subset of participants enrolled in a DAA treatment trial at a national referral hospital in Ho Chi Minh City, Vietnam. The goal of the qualitative study was to explore the experiences of care and treatment for these participants. Qualitative knowledge is vital to the process of developing public health programmes that effectively address barriers and promote access to care and treatment for people with HCV in Vietnam.

## 2. Materials and methods

### 2.1 Study context

Located in the South-East region of Asia, Vietnam is a middle-income country with a population of approximately 98 million people (World Population Review, 2021) with life expectancy increasing from 71 years to 76 years between 1990 and 2015 (Takashima et al., 2017; UNDP, 2016). Vietnam is a rapidly developing country with sustainable GDP rate growth between 6–7% annually within the last decade, resulting in GDP per capita rate in 2019 reaching $3,416 (STATISTA, 2021). Despite the negative impacts of the COVID-19 pandemic, Vietnam is one among a few countries that managed to advance its GDP by 2.91% in 2020 (Vietnam General Statistics Office, 2021a). This achievement could be explained by the significant low rate of COVID-19 infection cases in Vietnam during this period compared to other countries because of the swift responses, early actions and strong leadership of the government, as well as social mobilization (LTT Tran et al., 2021).

In Vietnam, the HCV seroprevalence is estimated to range from 1 to 4% (Berto et al., 2017; Quesada et al., 2015), while in specific settings like healthcare facilities or for dialysis patients, seroprevalence rates were reported to be as high as 26.6% (Berto et al., 2017; Dunford et al., 2012). In populations including people who inject drugs (PWID) and men who have sex with men (MSM), the seroprevalence was higher: for PWID, ranging from 52.8–58.3% or even up to 66% in some studies, and between 36.3–41.2% for MSM (Berto et al., 2017; Dunford et al., 2012; Rapoud et al., 2020). Results from studies indicate that the majority of HCV transmission is caused by using or sharing of non-sterile injecting equipment and/or blood transfusion practices, as well as other forms of therapeutic interventions that are done via intravenous approaches (Berto et al., 2017; Blach et al., 2017; Dunford et al., 2012).

In 2015, Vietnam established the National Action Plan on Viral Hepatitis, which focused on decreasing transmission and enhancing access to cost-effective prevention and treatment services through promotion of scientific research, advocacy for policy changes, and increasing collective action towards elimination (World Health Organization, 2016b). By the end of 2018, the health insurance drug list was updated to include DAAs (Ministry of Health Vietnam, 2018) and there was a push for a reduction of DAA costs and increased access (Due et al., 2020). However, “low cost” does not equate to “affordable”. Nguyen et al. (2018) assessed the willingness to pay for testing and antiviral treatment of people with HCV in Hai Duong, Vietnam and found that 440 USD was the maximum amount that participants were willing to pay but the most economical price for DAAs therapy, including diagnosis examination, was more than double at a minimum of 980 USD, with health insurance and health status restrictions (Due et al., 2020; Nguyen et al., 2018). More recently, a Clinton Health Access Initiative report published in 2021 revealed that 12-weeks of treatment in Vietnam using Daclatasivir and Sofosbuvir would cost 1022 USD, which was significantly higher than other countries in the region (e.g., Myanmar 150 USD, Indonesia 106 USD, Cambodia 75 USD, India 39 USD; Clinton Health Access Initiative, 2021) and unaffordable as the average yearly expenditure for healthcare was only 129 USD (Vietnam General Statistics Office, 2020; Vietnam Ministry of Health, 2021). These factors combined significantly hindered people’s access to DAA treatment.

### 2.2 Participants, recruitment, sampling

We recruited participants from a clinical trial focused on DAA treatment at the Hospital for Tropical Diseases in Ho Chi Minh City, Vietnam. We used a mixture of quota and purposive sampling to select participants that would maximize the range of experiences. First, for quota sampling, we aimed to enrol between 50–60% of the participants from stratum A (less severe disease) and the remaining 40–50% from stratum B (more severe diseases) of the clinical trial. We thought that the care and treatment experiences of those with varying severity of disease would be different. We also selected participants purposively based on data from a survey collected prior to the clinical trial, including age, gender, location, and potential risk factors. The sample size was between 18–24 total participants.

### 2.3 Data collection and analysis

We used multiple types of interviews in this study: in-depth (IDI), walk and talk, and home visits conducted with participants between March 2019—January 2020 in southern Vietnam. We also conducted observations in both the clinical trial and the hospital outpatient clinic waiting rooms on a regular basis during the same period. Two research assistants (RAs), who were not part of the clinical trial team, conducted all interviews with participants in Vietnamese. Participants were recruited and invited to have the three interviews at three separate time points: the first interview was conducted on the first day of enrolment (Day 0) into the clinical trial (i.e., first day of taking DAAs), the second interviews were conducted after participants completed DAA treatment within the participants’ home and surrounding areas, and the third interview was then conducted when the participants came to the clinical trial site for the last follow-up study visit in the clinic, which was 12 weeks after they completed of DAA treatment. Data collected from interviews were taken at different locations and at separate time points so that we could explore the progress and potential changes in experiences, perceptions and behaviours of participants towards the HCV, care-seeking and manoeuvring social support to access cure. Interviews that took place in the hospital were held one on one and for visits that took place outside of the hospital (e.g., the second interviews: home visit interviews, walk and talk interviews), there were always two RA present. Participants were given DAA medication at no cost and also received reimbursement for their participation in the trial, as well as in the qualitative research component. We audio recorded all interview sessions, including the walk and talk interview. We transcribed all interviews into Vietnamese and translated into English.

In the first and third interviews, we used a semi-structured interview guide with open-ended questions. The interview questions were piloted with members of community advisory groups (CAG) for hepatitis C for clarity and to determine the most appropriate terms for “cure” and terminologies for ’hepatitis C’/’liver diseases’/’viral hepatitis’ more broadly. We created summaries of the first interviews to create follow-up questions for the subsequent interviews. During the mobile interview, the discourse and the direction of the interview was meant to be participant led (Chang, 2017), therefore we did not use interview guides. However, in the home visit portion, we followed up on questions that arose from the summaries of the first interviews. For observations, the RA used hand written notes to document the informal discussions and observations that occurred in the clinic areas. We also used fieldnotes to document aspects of the walk and talk and home visit interviews, as not everything was captured on a voice recorder.

We used a revised version of thematic analysis by Braun & Clarke (2006) to analyse the data. We created initial process codes after reviewing all data and examining the first interview summaries in detail, we then piloted the codebook on three full participants’ transcripts (a total of 9 separate interviews) and made revisions. Finally, we applied the codes to the full set of transcripts adding new codes as needed (Braun & Clarke, 2006; Saldana, 2016). The study investigator and RA coded the data in either English or Vietnamese, respectively, and compared the results for a sub-set of the transcripts. The remaining transcripts were coded in Vietnamese.

We presented preliminary results at a conference in Ho Chi Minh City in December 2019 that was attended by health care professionals and public health experts and obtained advice and comments on the interpretation of results. We also verified findings with the study investigators from the HTD and members of the CAG. The Biomedical Research Ethics Committee at Hospital of Tropical Diseases (Vietnam) and Oxford Tropical Research Ethics Committee reviewed and approved the protocol. All participants provided separate written consent prior to joining the study.

## 3. Results

We conducted 14 observation sessions in the clinical trial waiting room, as well as five observation sessions in the outpatient department waiting area. An observation session in the clinical trial waiting room typically lasted one hour, while observations in the outpatient department waiting room ranged from 20 to 45 minutes for each session. These sessions enabled us to observe participants seeking care, communicating with each other, and ultimately allowed us to gain a better understanding of the wider study context.

A total of 20 participants provided consent to participate in the qualitative study and completed the first interview. Of those, one participant was withdrawn from the clinical trial by study doctors and therefore also withdrew from the qualitative component after the first interview, 16 completed the second interview session (including either a mobile interview, a home interview or both), and 18 completed the last study visit interview. Overall, there were 12 females and eight males, with 11 participants (55%) from stratum A of the clinical trial. For highest level of education completed, seven (35%) completed primary and nine (40%) completed secondary school (i.e., grades 6 to 9 for ages 12–15) , three (15%) completed high school (i.e., grades 10 to 12 for ages 16–18). One person did not attend school. Half of participants (10/20 or 50%) earned less than 5 million VND (approximately 215 USD) per month or had no income. These income groups correspond with the low to middle range of average monthly income in Vietnam, which was approximately 5.7 million VND (approximately 245 USD; Vietnam General Statistics Office, 2021b). Participants came from 13 provinces in the southern part of Vietnam. See, Table I.

**Table I.** Demographic information for qualitative research participants.

Sex	Age	Marital Status	Province	Occupation	Income (per month)	Education
F	1980	Married	Binh Duong	Manicurist	Under 5 M	Primary School
F	1965	Married	Binh Duong	Housewife	10 M-20 M	Secondary School
F	1952	Widowed	An Giang	Farmer	Under 5 M	Primary School
M	1990	Unmarried	Dong Thap	Farmer	5 M-10 M	Secondary School
M	1952	Married	Lam Dong	Farmer	5 M-10 M	Secondary School
F	1954	Married	Tay Ninh	Housewife	Under 5 M	Primary School
M	1977	Married	Dak Nong	Farmer	5 M-10 M	Secondary School
M	1961	Married	Ben Tre	Farmer	Under 5 M	High School
F	1959	Married	Tien Giang	Self-employment	5 M-10 M	Secondary School
F	1980	Divorced	Long An	Factory Worker	5 M-10 M	Secondary School
M	1962	Married	Tp HCM	Retired	5 M-10 M	High School
M	1974	Married	Ben Tre	Farmer	UNKNOWN	Secondary School
F	1959	Married	Vinh Long	Farmer	Under 5 M	None
M	1959	Married	Ca Mau	Farmer	Under 5 M	Primary School
F	1957	Married	Binh Thuan	Farmer	Under 5 M	Secondary School
F	1947	Widowed	Vinh Long	Housewife	5 M-10 M	High School
M	1973	Married	Binh Thuan	Fisherman	Under 5 M	Primary School
F	1965	Married	Binh Duong	Food Vendor	5 M-10 M	Secondary School
F	1948	Married	Tp HCM	Housewife	No income	Primary School
F	1955	Widowed	Tien Giang	Housewife	No income	Primary School

The first interviews lasted between 32–72 minutes each, while the second visit interviews were often much longer (i.e., between 22–66 minutes for the recorded walk and talk and 28–74 minutes for the recorded home visit interview), and the last visit interviews ranged from 24–86 minutes.

Based on our analysis, there were four major themes: navigating uncertainty, proactivity in the face of challenges, living in fear with faith, and dynamic support systems. Each is described in detail below.

### 3.1 Navigating uncertainty

I don’t know why. It [viral hepatitis] just came out of my body … right, my immune system is very weak, that’s why it [virus] was easy to intrude [into my body]. (B014 – LV)

The perceptions of uncertainty related to viral hepatitis were multi-faceted, including uncertainties and doubts about disease acquisition, capacity of local health systems and of traditional healers and medicine, and other doubts stemming from obtaining different information from a variety of sources.

#### 3.1.1. Disease acquisition (and re-acquisition)

The majority of participants had no clear idea why or how they were infected with HCV but many had potential explanations. The reasons revolved around aspects of their physical body: their strength of their immune system (e.g., too weak), they ate food with toxins, or lifestyle choices (e.g., drinking alcohol, lack of sleep). The explanations often included how the above factors led HCV to break out from their own bodies, as if it was lying dormant waiting for an opportunity.

*P:* *I stayed up late. I drank beer. My shift was from 7pm to 7am. These things gave me liver disease. I stayed up late so my liver could not do its job. After 11pm, [when we sleep] the liver has to filter and release toxins out of our bodies. But that time I was still awake. That’s why my liver was affected. Those two reasons. […]*

*R:* *but why do you think you caught this virus?*

*P:* *I think it’s because of my wrong diet. I drank. So the virus attacked me. […]*

*R:* *but where does this virus come from?*

*P:* *I think it’s in the food. I ate and got it. First, the food was not hygienic. Secondly, it was intoxicated. I ate it in … the toxins could not escape but piled up. Day after day, it became the root of this disease. I think so.*

*R:* *and it waits until you were weak …*

*P:* *that it beat me up, it broke out. It attacked me. (A020—HV)*

Others grappled with how they were infected by listing potential routes of infection. At a home visit, the participant, his brother, and his mother were discussing how the participant could have acquired HCV, again attributing disease causation to toxins or polluted/dirty spaces.

*P’s mother:* *[in] the whole family, nobody has this disease, only him [participant]*

*P’s brother:* *I am afraid he has it because he sprays pesticides*

*P:* *I work on garden so I have to use pesticide*

*P’s mother:* *Using pesticide for a long time brings consequences. So now he is no longer using it. He stays at home. […]*

*P:* *I don’t come to polluted places.*

*R:* *So, you mean the infection zone is where they breed?*

*P & P’s mother:* *yeah.*

*P:* *Where they breed and graze pigs and the crowded places. Complicated.*

*P’s mother:* *dirty and such. He works at clean areas. (A011—HV)*

There were also uncertainties regarding why hepatitis C would “return” for some people (i.e., unsuccessful treatment with interferon-based treatments or potentially reinfection after successful cure) but not others or how various forms of medication would impact the person more broadly.

I don’t know, I see the disease returns to some people, others are cured completely […] somebody did the injections that costed them millions but the disease still came back […] I heard that the injection is 50-60% guaranteed that the disease will not come back but not 100% […] that’s why I was confused, I didn’t dare to try it (laughs) it costs money to do the injection but it’s still not guaranteed, not to mention it can make the status worse. (B018 – LV)

#### 3.1.2. Capacity of local health systems

There were also uncertainties and doubts regarding the capacity of the local health systems to manage more severe diseases (e.g., viral hepatitis), as well the effectiveness of the medication covered by the national health insurance.

I have health insurance but the doctor is not qualified. They are not as good as doctors here [at the national hospital]. They are not as skillful … I was examined there [in local hospital] so I know. Many sick people had to come here [city hospitals but not specified which one]. People from the North and the Central [Vietnam]. Here [at big hospitals in city] they feel more assured … It [the overall upset and disappointed feeling when she had to travel here to be treated] sucks. To be honest, if I have to die, I would rather die here [at hospital in city, rather than being treated inefficiently and died at local hospital]. (B022 – Day 0)

The local hospitals were not trusted for more severe diseases. This was often followed up with the broader perception that speciality doctors were only based at the larger hospitals and not at the local hospitals.

Now if people are sick, no matter what disease, they will come to the city … . they all come to the city. There are 15-20 buses that take people to the city for health check-ups. Yeah now, frankly speaking, all good doctors come to city hospital and no good doctors stay at provincial hospitals. (A033 HV – LV)

This lack of trust in the expertise and efficiency of treatment provided by local care facilities caused people to move to central-line hospitals in big cities for more “proper care”, which often resulted in a different set of challenges. Some participants reported that even if they wanted to be discharged and move to the city hospital, the local hospital did not grant them the approval easily; instead, they insisted they could treat HCV. While for others, when they finally arrived at the city hospital, they encountered another major challenge related to the logistics of using health insurance. This challenges of “switching lines” of their health insurance in order to be able to use it in the city hospitals are described in more detail in subsequent sections. A few participants reported they ended up paying more for private care and “service medication” since they did not believe in the efficacy of medications covered by health insurance anyway. This idea was partially because local hospitals did not have DAAs at that time, only maintenance medication that was not meant to cure HCV. Participants spoke about how these medications were not an effective cure.

Because every time I went to the hospital at HT [a province] to use my health insurance, they gave me a big bag of pills that I took but never felt better. Too many pills. I didn’t know what were those meds but it never made me feel better … if I don’t have money, I would have to die on my death bed because hospital at HT couldn’t cure. (A033 HV – LV)

#### 3.1.3. Capacity of traditional medication and local healers

In addition to obtaining care and treatment at the local health centres and hospitals, several participants relied on traditional medication at some point during their care seeking journey. While many participants were told that traditional medications would not cure HCV, they still opted to take it because they felt they had no other choice or were not aware of its lack of effectiveness at the time that they took it. The capacity of the traditional medication was also uncertain and many cases uneffective, casting more doubts, confusion and hopelessness in participants’ minds. However, most participants resorted to traditional interventions initially due to its low costs and convenience.

*P:* *it’s hard to make money in the countryside … and travelling to city costs a lot of money … so it’s inconvenient. When they finally come, it’s already severe. That’s why people say they “feed the disease.” At first, they would not treat it, they wait until it gets worse, it makes them so tired that they will go. Most people in countryside are like that.*

*R:* *then how can they have some money to go to city?*

*P:* *they cannot, so they take Southern meds [one type of traditional non-pharmaceutical medications in Vietnam from the Southern region]. To live or to die, they take risk.*

*R:* *they have no other option, don’t they?*

*P:* *many people don’t even have money for travelling bus […] when they finally receive their diagnosis, they have to sell their stuffs or they take Southern meds. […] they have to try every way. (A014—HV)*

Another reason why participants took traditional medication was because often HCV is lumped with liver diseases more broadly (e.g., when confirmation of HCV was not possible). Others received good feedback and recommendations from their acquaintances. For liver disease, there was a common belief that the liver would get better if it was cooled down, which can be achieved with some herbs, roots, and leaves. While some participants tried this method as well, others still considered it weaker than Western medications.

He said [Southern meds] would cure but I was afraid it wouldn’t, it wouldn’t cure completely. Taking Southern meds can cool down my liver, make me feel better … But this liver disease is more severe. I am afraid Southern meds are too weak to be able to cure. (A035 – Day 0)

#### 3.1.4. Uncertainty from multiple information sources

For some participants, receiving multiple suggestions about what to do led them to try everything to find out what option might work. For others, having too much information led to more confusion and hesitancy. Therefore, they ended up not doing anything because they doubted its efficacy and would still cost a lot of money.

In the beginning, I went to AP [local hospital] where they told me I had Hep C. Severe virus. I was scared, thinking that it would turn to Hep B […] Then my wife told me to come to HH [city hospital] and it cost me almost 100 million [VND]. But it didn’t get any better […] I was sad. I am a fisherman. I work hard but all money I earn has to be spent on medications […] some people told me to try YD […] The doctor at YD said my status was normal, nothing. I didn’t know who should I trust (A033 – Day 0)

In sum, the uncertainty about the biology of HCV, inconsistencies regarding the quality and effectiveness of care and treatment, and doubts about local health systems led some participants to become proactive in care-seeking while also learning to live with fear and faith simultaneously. Both will be described in the next two sections.

### 3.2 Proactivity in the face of challenges

Despite, or perhaps because of, the uncertainties and doubts associated with HCV care seeking and treatment, participants became proactive while simultaneously facing multiple barriers and challenges. First, participants often faced difficulties adhering to routine doctor appointments and taking medication as scheduled. One major hurdle was financing the trip to the city hospital but participants found ways to manage with limited resources, as well as prepared for encouters with unexpected non-medical costs, in a city in which they were not familiar.

The most difficult thing is money, no money. The time when I still went to the hospital, every time when it came to my day of going, I was very worried … about money. I tried my best to save up days before my check-up day. Because I didn’t know how much it would cost exactly so I borrow some more [money] just in case. Then I will work to pay it back […] it’s hard to make money though. At this age, I am old, I cannot make any money. And my children, they also don’t have much. They have to pay their rent, their children’s school fees. (A035 – LV)

[about returning home after check-up] have to be quick, run to the bus so have no time for eating … when they finally sit on the bus, that [is when] people will start to eat, but just a bit. If they are late for this bus, where should they sleep? It costs money to rent a room and also people in the countryside they don’t understand the English word ‘hotel’ … well, it should have been written in Vietnamese so they can understand. Many people never came to city before or don’t have any relatives living in city to ask. Plus, people in the countryside are usually told that people at city will charge them higher for food so they don’t dare to eat anything here. If they do, they only buy a bread, a cup of water, that’s it. (A034 – LV)

As mentioned previously, participants spoke about the difficulties with using health insurance and how being unsuccessful with the health insurance process usually meant more financial burden. To avoid this lengthy and often exhausting process, many people (or their family) paid directly for the care.

I had to ask for referral documents so that I could go to the city hospital and use my health insurance. But there were times they didn’t give me such documents. They told me they could still treat my liver [disease] so they didn’t let me go. […] then my son told me to go service. 2 million for every 3 months … it has been 7 years like that […] for medications and bus tickets. Service. Taking those meds so I could work. Before that I couldn’t. [I was] very very tired. I couldn’t even herd my chickens … […] everybody told me I looked like I came back from the death [laugh]. (B011 – HV)

However, some participants challenged the local hospital staff until they could go to the central-line hospital despite the fact that they would have difficulties of “switching lines” for their health insurance in order to use it in the city hospital.

They [local hospital] didn’t do fibro scan or measure the viral load. They only did blood check to see liver enzymes. And did ultrasound on my liver. That’s all. But it was very hard to switch health insurance line [asking for transferring document]. They didn’t let me go. They said: ‘meds for liver can be found everywhere’. Then I had to fight with them. I told them I had been at this hospital for a long time. When I was at CR [a city hospital], the doctor there could measure my viral load. But at this hospital [local hospital], they couldn’t. I told them if they could [measure viral load], I wouldn’t have to go. I fought a lot. Then they finally let me go. (B016 – Day 0)

To switch lines, I had to go to local hospital to ask for it, then bring the referral document back home so that the next day I can depart early in the morning, then brought this document with me to the city [hospital]. This document could only be used for 3 trips then I had to renew it. Going back and forth, back and forth … (B016 –fieldnotes, outside the trial clinic)

Other challenges included having other priorities taking precedence over the HCV care and treatment, including work, caring for sick family members, and co-morbid health conditions. Mental health challenges were also reported among a few participants, often revealing feelings of hopelessness about illnesses and other difficulties that put them under tremendous pressure. Part of the narrative of life was to be cured from hepatitis and move forward.

I was planning to die by car accident but I was not brave enough, thought what if I couldn’t die but become disable? It would make my children suffer. I wanted to jump to the ground but I was afraid of becoming disabled also … afraid my children would have to take care of me. So, at the end, I thought to myself I have to be brave, not to die but to live, [to get cured] and pay my debt. (B014 – HV)

Despite these challenges, participants continued to look for alternative or complementary strategies. First, and as previously mentioned, many participants consulted traditional/village healers and took traditional medications before, during, or after using Western medicine. According to participants, these traditional medications were usually given away for free or at very cheap prices, following the diagnosis of the village healers who lived close or in nearby provinces. If the healers lived in other provinces, participants would travel by bus in small groups from their the villages. At the facility where the traditional care was provided, there were medications that included a wide variety of herbs and plants which were believed to cure not only viral hepatitis but other diseases. When participants arrived, they were given free meals in a communal room before lining up to be examined and bringing home the free herbal medications that were collected, categorized and packed to small portions in advance by the facilities’ volunteers. Information about herbal medications and how it could serve as a cure were circulated in participant’s community in a form of advice that was based on real-life experiences and passed down for generations.

They took six or seven kinds of them [Southern meds] and mixed it together. Each time, they gave me 30 bags of it so I could take it for one month […] I took it every day […] boiled it every day and poured it into the bottle and brought it to the field with me. Until I could no longer work on the field and I stayed home, but still drank it […] I took in for three or four months but my disease stayed the same. These meds were free. People gave it for free like charity. Many people came to that ‘traditional med station’ to receive it. Each day, thousands of people. […] (B011 – HV)

Participants often knew that taking traditional medication was not a cure for HCV but trying these interventions was one reaction to the situation.

Another form of treatment included self-comforting and/or changing their lifestyle after diagnosis.

I don’t think much. Because now I know what my disease is so I have to treat it, think that one day it will be over. Yeah, I think I have to be optimistic, not pessimistic […] so I can be cured fast. Yeah. I and my wife do exercise every day. Healthy diet. (A020 – Day 0)

Finally, joining a clinical trial was seen as both an opportunity and a risk: a proactive decision and/or having no other choices. For those who fully believed in the study medication and study doctors, they felt lucky to be “chosen” to participate in the study. They felt they were in debt to the study as they considered being enrolled to the trial to get free treatment was a rare chance, a silver lining. For those who did not fully believe in the study, they viewed joining the study as taking a risk, but because they “had no other choice”, they took the risk and had faith. For some, faith in the treatment was necessary for the treatment to work but it was not always an easy choice to make.

I am not worried because I know I couldn’t afford treatment so I … it’s like [sobbing] I take risk. If the meds are ‘suitable’, luck will come. It also helps me in term of finances. If I count on my financial condition, I don’t know when will I have the chance to take treatment. (B016 – Day 0)

### 3.3 Living in fear with faith

There were many things to fear, stemming from uncertainty and doubts, but also from the lack of financial access to care and treatment. The fear of the out-of-pocket treatment fees coupled with the fear of liver disease progressing to cirrhosis and then liver cancer led to complex situations where people had to make difficult choices. If one had no money for treatment, then their disease progressed forcing them to stay home and unable to earn their living in the long term. Even if they could financially afford their treatment, another major fear was to spend all their savings on treatment only to not achieve cure and/or become re-infected.

This liver disease is dangerous. I saw other people … I am scared … don’t know if it gets worse, how can I afford treatment? If it gets worse, I have to stay in bed and cannot make any money. (A027 – HV)

Since participants tended to lump HCV under the broad category of “liver disease” and liver disease is believed to result in fatality ultimately, they feared that their status was rapidly progressing, heading towards cirrhosis and/or liver cancer. This fear was amplified, especially when treatment at the hospital was financially (and otherwise), beyond their reach.

Many people are scared. Because they said cirrhosis … many people, they said [Hep C] usually turns to cirrhosis. They said year 2000 was when many people were diagnosed with this disease. They were afraid it’s liver cancer. Cancer is death. They don’t know where it came from [cancer]. They didn’t know. They struggled … especially in the countryside. Many of them. (A034 – Day 0)

Even if they were able to treat the viral hepatitis with DAAs by joining clinical trial, they were also fearful of the new drugs’ potential side effects.

The doctor told me that now they have modern meds [DAA] […] I told him I don’t have money for that so he introduced me to come here [study] but I refused […] I was afraid taking this med [study medication] would knock me down, would kill me (A028 – Day 0)

But people also coupled the fear with faith.

I thought I was like … offering myself to God […] not sure it would be good or bad [joining trial] so I didn’t tell my kids. […] I thought to myself joining this study was like having luck, I thought it that way so I could be confident. I comforted myself. I counted on my luck. You see, I couldn’t afford target meds and left my disease lingered did more harm than good, so I had to count on my luck. (B016 – HV)

The notion of fear and faith were intertwined within the pathway to cure. For example, the decision to join the trial was often very difficult as they were not certain about its outcome. However, they still decided to have faith in it and “counted on their luck” to comfort themselves. As mentioned above, they considered taking medications from this trial as both an opportunity and a risk; and “taking risks” in this situation not only demonstrated participants’ difficult setting when thay had no other way to be treated besides joining the trial; “taking risks” also reflected participant’s strong believe in *fate*. They felt lucky to be invited or “chosen” to participate the trial. Being able to join was a blessing, and it was fate that they were blessed. Above all, the aspect of “taking risk” was their believe in karma and themselves, as explained when an participant elaborated about “can tu”.

When I joined this study, people said I have ‘can tu’. ‘Can tu’ is blessing. To be able to join this study, one needs to be blessed. They said I must have been blessed my ancestors, really blessed to be recruited. That’s fate. Thanks to the doctor that introduced me to this study otherwise I couldn’t know. How could I? I couldn’t join even if I begged. I came to this hospital [HTD] for almost 1 year back and forth, until suddenly my doctor introduced me. If he didn’t introduce, how could I join?

Can tu is: in my previous life, I tried my best to live morally, to be a good person, to help other people … so [this life] I am blessed. […] They said I was saved by God but I told them I was saved by HTD, not God. (B014 – Day 0)

While people believed in fate, a notion that implies everything in life is predetermined, they still proactively sought out for treatment and tried their best to adhere to clinical visits and treatment. There was a general sense that many participants did not understand all the details about the recruitment process (e.g., inclusion and exclusion criteria) and thought that they were blessed to join and that the study staff wanted to help them gain access to medication. For those who clearly understood that they were enrolled in a clinical trial, they had belief in this study process, as they knew that the meds were tested prior to the trial. For others, they simply refused to join. There were also some participants who were fearful and refused to join at first but in the end, they still decide to “take risk and seize the chance” (B016—HV)

Another reason why participants decided to have faith in DAAs was they were all recruited from the outpatient department of the hospital so they easily made comparisons with the care and treatment offered there. Compared to the meds at the outpatient department, the DAAs were more costly, had a shorter treatment duration, which in their minds also meant the drugs were stronger, and were brought to them to by Western study doctors. Besides, these factors, participants believe that the drugs’ efficacy also had to depend on individual’s body—“matching” or “suitable” with the body and had to be taken with the right dosage on a regular routine so it could work.

If my body ‘accepts’ the med, it will be good. If it’s good meds, it will cure faster … I don’t know because I think all doctors are good doctors. They give diagnosis. But the meds have to be fit with our bodies … that’s why I say it’s about luck [laugh] meds are effective but it has to depend on our bodies. These two combined. And our diet. Every day we have to take the right portion of meds so it can be ‘qualified’ (A014 – HV)

When it finally worked, the sign of this medication’s efficiency on the body would then be manifested by participants’ ability to eat and sleep as “normal,” or as “before”.

I don’t know why I take this study meds just 2 bottles of it then I feel much better. I gain weights. I think I am cured. Because I feel better. Previously I have not yet taken it, I felt bad. After meal, I always had to go lay down on my hammock. (A027 – HV)

The meaning of “cure”, thus, played out in specific scenarios from daily life in which they attributed “cure” to the practical benefits that they experienced, such as the ability to get up, eat and walk around more easily, sleep well, work, earn money and take care of their families. Being cured meant they gained strength back, both physically and mentally, and stopped considering themselves a burden to their family, on the contrary, they became a healthy and productive member able to contribute. In sum, biomedically “cure” is defined by diminished viral load but for participants, cure meant more than a viral load result or a number on their medical file, but a guarantee for a better quality of life for their whole family.

### 3.4 Dynamic systems of support

My kids … when I went to the hospital, if I had money, I would use my money. But when I didn’t, my kids would gather and gave me some money. They gave me 50 thousand [about 2 USD], each of them … they don’t have much so they gathered and gave me their shares. All my kids are hard labor workers. (B011 – HV)

When participants received support, it came in several forms: pragmatic support (e.g., switching work shifts with colleagues to make the appointments), financial support, emotional support, and personal connections (e.g., with doctors to help explain). Although many participants’ and their families’ financial conditions were limited, most of the participants received support from their family, including emotional and financial support. For others, the support came in the form of advice, which they did not always follow.

Sometimes when I met my sisters in law, they said: ‘you should go to HTD because they have specialized department in liver. Try your best to take their treatment. Not so costly. Only a few million’ so the first time, I came with her. She had gone to HTD multiple times before so she knows. I followed her. (A035 – Day 0)

I don’t take it [Southern meds]. Many people at this hospital [patients who also came to check their livers] told me to try. It’s like one suggested another […] but I didn’t try. I thought how could Southern meds kill this virus? (B016 – Day 0)

Despite receiving various form of support from friends and family, some participants revealed they did not have a chance to obtain explanations about their diseases from the healthcare providers. For example, even though local doctors recommended that they go to HTD for treatment, there was no explanation about viral hepatitis, and the participants did not feel like they could ask for this support.

They didn’t say what is hepatitis C … I thought because doctors had too many patients waiting so they didn’t have time to explain … I couldn’t ask because it was very crowded, too crowded, people kept coming. I couldn’t ask. (A035 – Day 0)

Due to this lack of clear and succint information from healthcare providers, participants had to turn to other patients for advice, promoting a dynamic support system between patients and family members, and among patients.

They [healthcare providers] said nothing. Didn’t tell me if this [disease] is severe or not or how to treat it. They only gave me schedule for the next check-up […] I brought my files outside. I looked at it. I asked other people to have a look at my file for me. They were also patients waiting outside. They said with this diagnosis, I had to treat it, otherwise it would turn to cirrhosis. (B023 – Day 0)

It is worth noting that individual feelings of discrimination and/or stigma due to HCV was not a dominant theme in the narratives of participants, however, there were some complexities surrounding perceptions of HCV more broadly. The ways in which HCV is conceptualized and experienced will be further explored in a subsequent analysis.

## 4. Discussion

The care-seeking experiences of people with HCV are journeys of navigating and negotiating. In this study, the participants described how they manoeuvred through a complex web of uncertainty and proactivity, embedded in a dynamic system of support, often faced with both fear and faith. The feelings of uncertainty did not prevent participants from care seeking but on the contrary, they managed to overcome challenging conditions and proactively harnessed existing resources to find alternative solutions, including taking traditional non-pharmaceutical medications and adhering to treatments in a clinical trial.

Participants often faced financial burdens in the care and treatment pathways, which are well-documented challenges across many contexts. In Vietnam specifically, Berto et al. (2017) and Due et al. (2020) found people living with HCV could not start or complete their treatment due to financial issues caused by both direct medication fees as well as other non-medical costs (Berto et al., 2017; Due et al., 2020); while in Cameroon, even before DAAs were introduced into the setting, care and treatment costs for HCV were described as “catastrophic” (Chabrol et al., 2019). Considering participants could not immediately or easily change their financial situations, we found that they decided to change their approach to treatment. Many participants complimented seeking care from hospitals and medical centres with local village healers that were often more convenient and within their financial reach. This is not a surprising finding as in Vietnam, and elsewhere, medical pluralism is a common form of care seeking (Craig, 2002; AL Tran et al., 2020). Due to a lack of thorough explanations about their HCV diagnosis from healthcare providers, HCV as an illness was often included as part of a more general “liver disease” diagnosis. In the context of traditional medicine, participants discussed how herbal medicine would reduce the symptoms while cooling down the liver, falling in line with documented perceptions of Eastern medicine in Vietnam being associated with being slow, cooling, natural with Western medicine being fast, strong, chemical (Craig, 2002). The lack of concise information about viral hepatitis and its treatment, as well as the abundance of unconfirmed, and often incorrect information that came from different sources outside healthcare facilities, brought two scenarios: 1) hesitating to reach out for care because they did not know which treatments would work for them, or 2) trying every suggestion until they found what worked. Both scenarios often resulting in long care seeking pathways.

In this study, we also found the lack of consultation from healthcare providers at local level led to the lack of trust in local health system. The shortage of trust in local care, as explained by participants, also stemmed from unpleasant previous experiences with local hospitals and a belief that the local healthcare infrastructure did not have the capacity to diagnose and treat HCV. As a result, some participants advocated to move to hospitals located in the city which they believed had more capacity. The major challenges faced then was with the health insurance referral system. The national Direction of Healthcare Activities (DOHA) was established by Vietnam’s Ministry of Health, with the mission to promote technology transfer training from central-line hospitals to healthcare facilities at provincial and district-levels. The overall goal was to increase the lower level facilities’ capacity in order to reduce the burden at higher level hospitals (Takashima et al., 2017). Under DOHA, if the lower level clinics are being encouraged to care for more patients locally, then they would want to keep some patients locally (and refuse transfers) to demonstrate progress. Yet the local perception was that the clinics could not provide the necessary care and treatment, so the participants would face challenges to be referred to the next level. For those who did not receive the transfer or could not afford the travel to the city hospital, participants reported they preferred traditional medications and village healers instead of the local health centres.

Participants often consulted with family and acquaintances about what to do, meaning that they disclosed about their health status quite naturally. Most participants did not report feeling discriminated or stigmatized because of HCV. This finding was similar with what Serumondo et al. (2020) found in their research in Rwanda where there was a general lack of stigma and people encouraged each other to complete treatment (Serumondo et al., 2020). We also found that the clinical trial participants informally listened, gave advice and encouraged each other to adhere to treatment. In contrast to what we found, a study in Ho Chi Minh City conducted by Dam et al. (2016) revealed people living with HBV endured the feeling of guilt because they had “[put] others at risk” and created shame for their family due to infection (Dam et al., 2016). In our study, we found that many participants did not really differentiate between HCV and HBV but addressed them both as “benh gan” [liver disease]. Therefore, the feelings of guilt, instead of shame, stemmed from their self-realization that due to their infection of “benh gan”, they had become a financial burden to their family by either requiring financial support or their inability to contribute to family finances due to their health condition. Cure, thus, was defined as gaining back their strength so that they could work and reduce the burden from their family, both financially and emotionally. This finding is similar to that of Richmond et al. (2018) in Australia where patients revealed a sense of freedom after being cured. In both settings, participants treasured the cure as a way to end suffering and become a productive family member. The aspiration to be productive reaches into the community as well by participants advising to others where to be tested and diagnosed, and/or how to join the trial for treatment.

There was a gap between the researchers and participants regarding their perceptions of the clinical trial. Compared to what researchers and study staff saw the trial as research and identified people who joined the trial as study participants; the participants, on the contrary, regarded being able to enrol in the trial as a rare opportunity or a *blessing* to take free medication, which was also reported elsewhere. For example, a participant from a DAA treatment study in Rwanda reported he “waited for God to do miracles” to get him the curative treatment and that God led him to the study (Van Nuil et al., 2020). In our study, it is important to note that most participants did not know there was a trial that could give them free meds prior to enrolling; they reported that they only learned about the trial when doctors in the outpatient department at Hospital of Tropical Disease referred them to the trial. Therefore, getting free treatment with DAAs at the clinical trial was not an alternative care that they proactively sought in advance, but *accepting the risk* with both fear and faith to take on this alternative care when they saw the chance was instead the embodiment of proactivity. Being proactive did not equate to absolute confidence, but it proved how they were striving for health, despite major fear and uncertainty. A state of “not knowing” was also noted in Burkina Faso, where participants approached, defined, and ultimately turned “uncertainty” into concrete action (Giles-Vernick & Hejoaka, 2020). In our study, we witnessed a similar process among our participants where they successfully managed to transform their limited resources, their fear and uncertainty into a source of aspiration and faith, so as to enact their proactivity. Simply put, when consequences take shapes in mind, people act.

This study has several limitations. First, seven home-visits could not be conducted due to logistic reasons including travelling difficulties or because the participant was not comfortable for the researcher to visit their home. Second, we originally planned additional follow-up home-visits with 3–5 participants for 6–12 months after the completion of treatment but we removed this component due to COVID-19. Finally, we recruited participants solely from the clinical trial site so the narratives are limited to this specific group. We realized early in the study that key populations at risk for hepatitis C, including PWID and/or sex workers were underrepresented. We designed an additional study to explore barriers to care among these underserved groups.

The care-seeking experiences of people with HCV was characterized by a significant level of uncertainty caused by both the abundance of unconfirmed information as well as the shortage of clear and concise information about HCV and its treatment, and a general lack of trust from participants towards local healthcare facilities. However, these participants managed to navigate care and treatment. On one hand, we see journeys of fear since their limited financial conditions could not grant them the privilege to be sure about their decisions, but on the other hand, we see journeys of faith as they believed the cure would come to only those who worked hard for it. Within a dynamic support system, they received different forms of encouragement from others, while vice versa, they were also the ones who gave others advice. To conclude, this study provides a glimpse into the lived experienced of people with HCV in Vietnam, and highlights the importance of integrating cure into the pathways for those living with HCV.

## Data Availability

Data are available from the corresponding author upon reasonable request at https://www.oucru.org/wp-content/uploads/2017/04/OUCRU-Data-Request-Form-V1.1-090217.pdf.
